# Low-dose axitinib rechallenge with positive outcomes in a patient with metastatic renal cell carcinoma refractory to interferon α, sunitinib, axitinib, and nivolumab therapies: a case report

**DOI:** 10.1186/s13256-019-2041-8

**Published:** 2019-04-22

**Authors:** Masaki Murata, Yohei Ikeda, Go Hasegawa, Yuki Nakagawa, Tsutomu Nishiyama

**Affiliations:** 1 Department of Urology, Uonuma Institute of Community Medicine, Niigata University Medical and Dental Hospital, Urasa 4132, Minamiuonumashi, Niigata, 949-7302 Japan; 2 Department of Diagnostic Radiology, Uonuma Institute of Community Medicine, Niigata University Medical and Dental Hospital, Niigata, Japan; 3 Department of Pathology, Uonuma Institute of Community Medicine, Niigata University Medical and Dental Hospital, Niigata, Japan

**Keywords:** Metastatic renal cell carcinoma, Adverse events, Low-dose axitinib

## Abstract

**Background:**

There is no established treatment after failure of proven therapies for patients with metastatic renal cell carcinoma.

**Case presentation:**

A 66-year-old Japanese man with metastatic renal cell carcinoma became refractory to interferon α and sunitinib therapies. He started treatment with axitinib at 10 mg/day, and the dose was gradually tapered down to 4 mg/day because of intolerable adverse events. His metastatic lesions shrank; however, he could not continue due to the adverse events. He started fourth-line therapy with nivolumab; however, the metastatic lesions increased. Rechallenge with axitinib 4 mg/day was started, and the dose was reduced to 2 mg/day because of adverse events. Subsequently, the adverse events became controllable, and the metastatic lesions were maintained at reduced size.

**Conclusion:**

Therapeutic drug monitoring of axitinib could play an important role in the development of safe and effective therapeutic treatment and individualization of these medications.

## Introduction

Currently, metastatic renal cell carcinoma (mRCC) is treated with vascular endothelial growth factor (VEGF)-targeted agents, mammalian target of rapamycin (mTOR) inhibitors, and immuno-oncology (I-O) drugs including cytokines and immune checkpoint inhibitors such as nivolumab. Among these, VEGF receptor (VEGFR)-tyrosine kinase inhibitors (TKIs) and I-O drugs have been reported to have high therapeutic efficacy [1]. Furthermore, I-O drugs are recommended for early use for patients classed as intermediate or poor risk in the International Metastatic Renal Cell Carcinoma Database Consortium (IMDC) risk groups [1]. However, there is no established treatment after treatment failure of such agents [2–4]. We describe the case of a patient with mRCC refractory to interferon α, sunitinib, axitinib, and nivolumab therapies, who was treated with low-dose axitinib re-administration. A low-dose axitinib rechallenge down to 2 mg/day after nivolumab therapy resulted in positive outcomes with the metastases maintained at reduced size.

## Case report

**Fig. 1:**
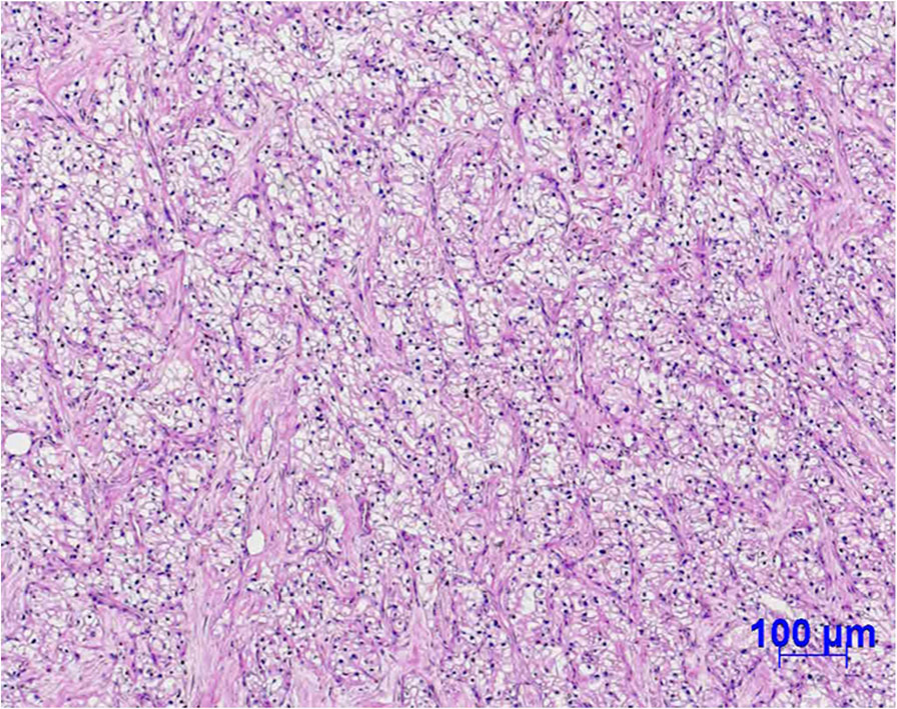
Pathological findings of original right renal tumor. Pathological diagnosis is clear cell carcinoma

**Fig. 2:**
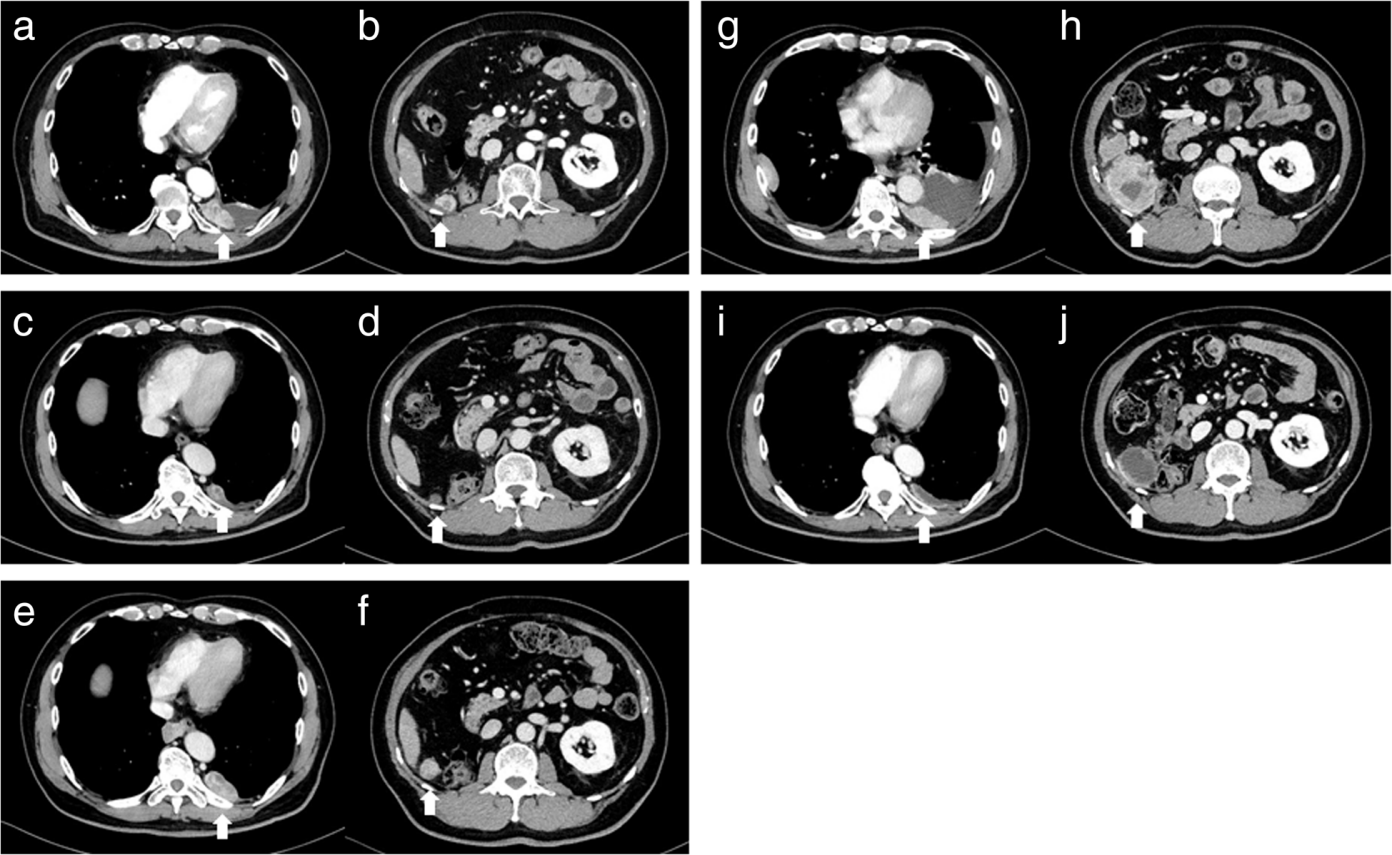
Computed tomography findings during therapeutic courses. **a**, **b** New metastases such as pleura, diaphragm, and the right paracolic gutter appeared after sunitinib treatment. **c**, **d** The metastases diminished in size and lung metastases maintained shrinking state after axitinib treatment. **e**, **f** The size of metastases increased again after discontinuation of axitinib. **g**, **h** Existing metastatic lesions were growing, peritoneal dissemination appeared in the pelvic region, and pleural effusion appeared after nivolumab treatment. **i**, **j** Metastases such as lung, pleura, diaphragm, and the right paracolic gutter diminished in size after low-dose axitinib (2 mg/day) treatment

**Fig. 3:**
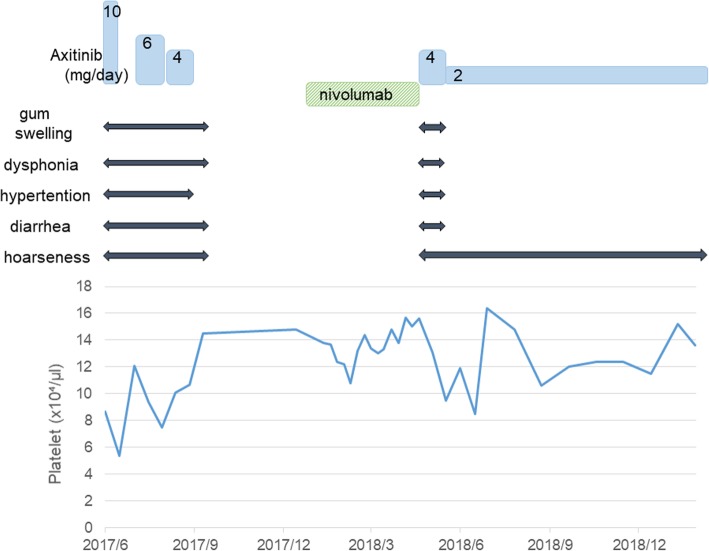
Time series showing adverse events for treatment with dosage changes of axitinib

A 66-year-old Japanese man who had no past medical or medication history complained of gross hematuria and visited a nearby hospital in October 2013. He had no habit of drinking alcohol or smoking tobacco. He was diagnosed as having a right renal tumor and underwent right nephrectomy laparoscopically. The pathological diagnosis was right renal cell carcinoma (RCC), clear cell carcinoma, pT1bN0M0, v1 (Fig. 1). One and half years later, lymph node swelling was detected at hepatic portal region and he underwent lymphadenectomy. The pathological diagnosis was a metastasis from RCC. Two years after diagnosis, he was suspected of lung metastases and started treatment with interferon α. Three years later, the multiple lung metastases grew larger and were determined as progression despite interferon α therapy. At this point, he was referred to our hospital in October 2016. There were no abnormalities on physical examination and his vital signs were normal. He started treatment with sunitinib 50 mg/day on a schedule of 4 weeks on treatment and 2 weeks off; however, adverse events including grade 3 thrombocytopenia (platelet count, 49,000/μL), gum swelling, and hoarseness became intolerable 2 weeks after starting sunitinib. Four weeks after cessation of sunitinib 50 mg/day, he was started on a dose of sunitinib 25 mg/day on a schedule of 2 weeks on and 1 week off. Computed tomography (CT) findings in January 2017 revealed that his lung metastases had shrunk; however, he continued to experience the same adverse events. Therefore, the dose of sunitinib was further reduced to 12.5 mg/day on a schedule of 2 weeks on and 1 week off. CT findings in May 2017 revealed new metastases in the pleura, diaphragm, and the right paracolic gutter (Fig. 2a, b). As a result, the treatment was changed from sunitinib to axitinib and he started treatment with axitinib at 10 mg/day; however, adverse events including gum swelling, dysphonia, hypertension, diarrhea, and thrombocytopenia became intolerable (Fig. 3). Two weeks after cessation of the drug, the dose of axitinib was gradually reduced from 6 mg/day to 4 mg/day. CT findings in September 2017 revealed the metastases had diminished in size and lung metastases were maintained at a diminished size (Fig. 2c, d); however, the adverse events could not be controlled and he discontinued axitinib treatment. His adverse events improved after discontinuation of axitinib; however, CT findings in December 2017 revealed the size of metastases had increased again (Fig. 2e, f). Consequently, he was started on fourth-line therapy with nivolumab (3 mg/kg every 2 weeks) and did not experience any adverse events. However, after he had received eight cycles of nivolumab, his metastatic lesions had grown, peritoneal dissemination appeared in his pelvic region, and pleural effusion appeared (Fig. 2g, h), so nivolumab was discontinued. After giving a detailed explanation of treatment options to our patient, he decided to rechallenge with axitinib 4 mg/day. However, adverse events including gum swelling and dysphonia became intolerable. After that, the dose of axitinib was reduced to 2 mg/day, and he experienced relief of adverse symptoms except for hoarseness. CT findings in August 2018 revealed metastases in lungs, pleura, diaphragm, and the right paracolic gutter had diminished in size (Fig. 2i, j). He has been continuously receiving a low dose of axitinib at 2 mg/day for 10 months with metastases maintained at reduced size.

## Discussion

We describe rechallenge with low-dose axitinib at 2 mg/day after nivolumab therapy and had positive outcomes with the metastases maintained at a reduced size. The adverse events of axitinib experienced by our patient were not controllable and it was discontinued even after gradual tapering to a dose of 4 mg/day.

RCC can be treated with curative intent surgery when diagnosed at a local stage; however, mRCC will progress in most cases and require systemic therapy. Early oncologic immunotherapies such as interleukin-2 and interferon-α were reported in the 1990s. They are non-specific cytokine therapies for RCC, and exert their effects by generating activation of T cell responses. The median survival time of Japanese patients with mRCC was approximately twice as long as that of patient in studies from North America or Europe [5]. Treatment of mRCC has evolved significantly over the past 20 years. Since 2005, the approved first-line treatment consists of TKIs such as sunitinib that target the VEGFRs. Second-line therapies include treatments targeting the VEGF pathway, and more recently immune checkpoint inhibitors such as nivolumab have been developed. Axitinib is a potent and selective inhibitor of VEGFRs 1, 2, and 3, approved for second-line therapy for advanced RCC. Recently, axitinib has been replaced by cabozantinib and nivolumab in the second-line setting in international guidelines, and the place of axitinib in therapy is therefore challenging. Axitinib is well tolerated, with the expected adverse events of VEGFR-TKIs. In most cases, these adverse events are manageable and reversible with dose adaptation or interruption and supportive care; however, the present case discontinued axitinib, even though the dose was gradually reduced to 4 mg/day because of adverse events such as gum swelling, dysphonia, and diarrhea. After rechallenge with axitinib at a dose reduced to 2 mg/day, there was relief of adverse symptoms except for hoarseness, and metastases of lungs, pleura, diaphragm, and the right paracolic gutter diminished in size. Japanese ethnicity is reported to be associated with decreased systemic clearance of axitinib, resulting in a higher exposure [6]. If our patient had been treated with a reduced dose of axitinib at 2 mg/day before the nivolumab therapy, the metastases could have been maintained at a diminished size. Recently, novel patterns of response and progression to immunotherapy such as durable response, pseudoprogression, hyperprogression, and dissociated response have been reported [7]. In our case, there may also have been a possibility of a durable response to nivolumab after pseudoprogression; however, we discontinued nivolumab because of disease progression. Standardized definitions and clear mechanisms of these responses have not been established and further clarification is needed [7]. Recently, axitinib in combination with an I-O drug was reported to have a striking effect on patients with mRCC [8, 9]. There may also have been the interactive effects of nivolumab and axitinib in our case.

## Conclusion

We described the case of a patient with mRCC treated with low-dose axitinib re-administration with positive outcomes after treatment failure of interferon α, sunitinib, axitinib, and nivolumab. Therapeutic drug monitoring of axitinib could play an important role in the development of safe and effective therapeutic treatment and individualization of these medications.

## Abbreviations

CT: Computed tomography
IMDC: International Metastatic Renal Cell Carcinoma Database Consortium
I-O: Immuno-oncology
mRCC: Metastatic renal cell carcinoma
mTOR: Mammalian target of rapamycin
RCC: Renal cell carcinoma
TKIs: Tyrosine kinase inhibitors
VEGF: Vascular endothelial growth factor
VEGFR: Vascular endothelial growth factor receptor
